# pGQL: A probabilistic graphical query language for gene expression time courses

**DOI:** 10.1186/1756-0381-4-9

**Published:** 2011-04-18

**Authors:** Ruben Schilling, Ivan G Costa, Alexander Schliep

**Affiliations:** 1 Max Planck Institute for Molecular Genetics, Department of Computational Biology, Ihnestr. 63-73, 14195 Berlin, Germany; 2 Center of Informatics, Federal University of Pernambuco, Av. Prof. Luiz Freire, s/n, Recife - Pernambuco - Brazil; 3 Department of Computer Science and BioMaPS Institute for Quantitative Biology, Rutgers, The State University of New Jersey, 110 Frelinghuysen Rd, Piscataway, NJ 08854-8019, USA

## Abstract

**Background:**

Timeboxes are graphical user interface widgets that were proposed to specify queries on time course data. As queries can be very easily defined, an exploratory analysis of time course data is greatly facilitated. While timeboxes are effective, they have no provisions for dealing with noisy data or data with fluctuations along the time axis, which is very common in many applications. In particular, this is true for the analysis of gene expression time courses, which are mostly derived from noisy microarray measurements at few unevenly sampled time points. From a data mining point of view the robust handling of data through a sound statistical model is of great importance.

**Results:**

We propose probabilistic timeboxes, which correspond to a specific class of Hidden Markov Models, that constitutes an established method in data mining. Since HMMs are a particular class of probabilistic graphical models we call our method Probabilistic Graphical Query Language. Its implementation was realized in the free software package pGQL. We evaluate its effectiveness in exploratory analysis on a yeast sporulation data set.

**Conclusions:**

We introduce a new approach to define dynamic, statistical queries on time course data. It supports an interactive exploration of reasonably large amounts of data and enables users without expert knowledge to specify fairly complex statistical models with ease. The expressivity of our approach is by its statistical nature greater and more robust with respect to amplitude and frequency fluctuation than the prior, deterministic timeboxes.

## Background

**Figure 1:**
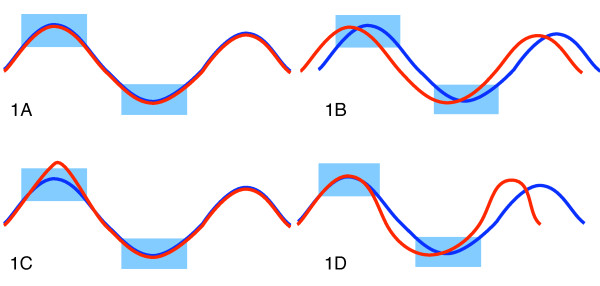
**Deterministic queries under the effect of common characteristics of time courses**. In (a) both time courses are in the result set of the deterministic query interpretation. For (b), (c) and (d) the red time course is not included in the result set. In (b) a phase shift has moved the red time course out of the query's scope. In (c) an outlier prevents the red time course from being included. In (d) a variation in the speed at which values are taken prevents the red time course from matching the deterministic query interpretation. See the Results section on how deterministic timebox queries are evaluated.

The analysis of gene expression time courses e.g. from DNA microarrays is crucial in understanding dynamical biological processes such as cell cycle, cell development and cell response to external stimuli. Common data sets consist of 5-30 time points and hundreds or thousands of genes [1,2]. In the beginning investigators often explore their data by querying for certain qualitative and quantitative behaviors as an informative visual inspection of multivariate time points is indeed difficult. We define *querying *here as the evaluation of a set of conditions on time courses. The result of a query is a score for each time course that can be used to select a subset of time courses exposing behavior specified through the conditions. Among the characteristics of time courses is their variation in speed (cf. Figure 1d), i.e. delay of similar observations inducing uncertainty about exact time periods where measurements are expected to happen and phase shifts (c.f. Figure 1b). Generally, missing values, noise and outliers (cf. Figure 1c) can commonly be expected. In particular, gene expression time courses have only very few, unevenly sampled time points [3], in contrast to temporal data from other domains such as finance or multimedia. Intuitive software tools which support interactive formulation of query parameters by non-experts can foster wider and more flexible usage of time course data analysis tools by scientists and analysts in a range of disciplines. Yet an open research problem is finding a robust method to interactively query short time courses for specific qualitative and quantitative behavior.

Previously, timeboxes--specifying minimal and maximal values for a continuous range of time points--were proposed [4] to select time courses of interest. Timeboxes are rectangular, graphical user interface (GUI) widgets, that are embedded in a graphical time course data display. However the method is not robust to noise, temporal or amplitude variations (c.f. Figure 1) common in typical data sets and does not attempt to model them. We previously implemented a method called GQL for analyzing time course data that used graphical sliders to specify parameters of densities used in a HMM [5,6]. The tool lacked however an intuitive relationship between the query parameters and the data.

We show that timeboxes have a natural interpretation as stochastic, piece-wise linear functions, or linear Hidden Markov Models (HMM) [7]. Their statistical interpretation allows for more flexible and meaningful queries, in particular in the context of analyzing biological data. We refer to our method as Probabilistic Graphical Query Language (pGQL), as HMMs are a specific class of (probabilistic) graphical models [8]. The visual query tool implementing our model allows users to define graphical model parameters; ranking time-courses by likelihood under that model and using a cut-off on the likelihood deter-mines the query result. Note that this is in contrast to the definition of query languages in the field of in-formation science, where graphical query languages are formal languages for querying graphs for graph-theoretical properties and/or attributes of nodes and edges. Although it is not possible to phrase all deterministic queries within pGQL, such as e.g. finding time courses whose values drop by 50% within a time interval or finding time courses for which there exists at least one time period, where they fluctuate by at most some quantity. However it is also not possible to phrase all probabilistic queries in a deterministic query system. The queries you can ask in pGQL are based on expected values and standard deviations. We demonstrate the effectiveness of probabilistic queries on a yeast sporulation data set using our implementation of the probabilistic time-boxes (pGQL). We discuss how our approach contrasts with deterministic timeboxes and identify impact our method has for practitioners.

## Results

### Algorithm

In the following we will refer to a set time courses as *O *= {*o_i_*}, 1 ≤ *i *≤ *N*, where each time course  takes values  for times *t *∈ {1, ..., *T*}.

#### Timeboxes

Let us start reviewing the definition of [4] for timeboxes and their queries formally:

A timebox *b_j _*is defined by the 4-tuple , where  and . For a given set of time courses *O *= {*o_i_*}, where 1 ≤ *i *≤ *N*,  and *d *is the number of time points observed, a time courses *o_i _*satisfies a query *b_j _*only if  for .

**Figure 2:**
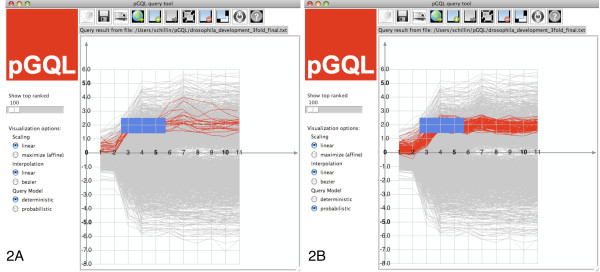
**Comparison of deterministic and probabilistic timeboxes**. (a) Query evaluation using a deterministic timebox (b) Query evaluation using probabilistic timeboxes The deterministic query in (a) does not contain all time courses showing the trend as defined by the graphical query and even contains time courses that do not follow the trend outside of the specified time interval. The corresponding probabilistic query in (b) however does not have such shortcomings and produces a much more consistent result on this real data set.

In Figure 2a, we display all time courses whose values lie in the interval [*x*^-^, *x*^+^] for all time points in the time frame . That is, for a given time-box  and set of time courses *O *= {*o_i_*}, where , a query is defined as(1)

Naturally the query operation can be easily extended for multiple timeboxes {*b*_1_, ..., *b_j_*, *b*_*j*+1_, ..., *b_q_*} where  holds ∀*j *∈ [1, *q*]. In this case, a time course satisfies the query only if , which is the intersection of individual queries: *Q*(*b*_1_, ..., *b_q_*) = ∩*_j_Q*(*b_j_*).

#### HMMs and Probabilistic Timeboxes

A HMM can be seen as a probabilistic function of a Markov chain [9] and is fully determined through the specification of its states *S*, the probability of starting in *s_i_*, the transition probability *π_ij _*from state *s_i _*to *s_j _*and the emission densities see [7] for details on Hidden Markov Models.

**Figure 3:**
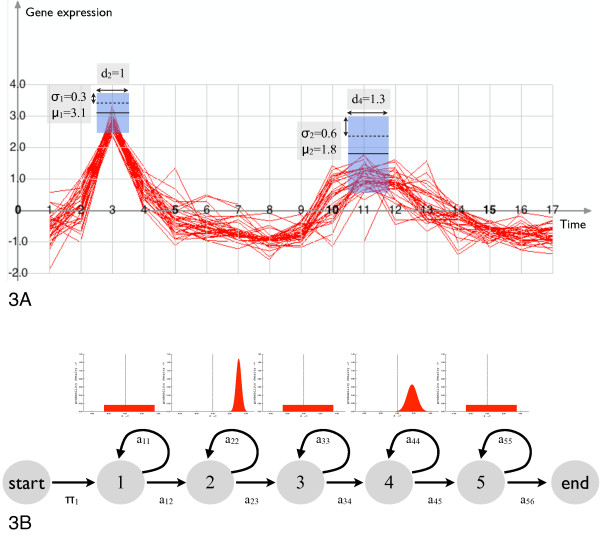
**Graphical Query and its interpretation as lHMM**. Example of the relation of a probabilistic timebox query (Fig. 3A) to its state model in the corresponding lHMM (Fig. 3B). In Fig. 3A the central black bar within each blue PTB represent the mean value, while a quarter of each PTBs height represents the variance. In Fig. 3B the states 2 and 4 correspond to the blue PTBs in Fig. 3A. When we compare Fig. 3A with Fig. 3B we see, that pGQL inserts automatically hidden states with a uniform distribution in areas where no blue PTB has been placed.

A particular HMM [2,9] specialized for time course analysis is the following. The observed states correspond to the user drawn boxes, the hidden states to (graphically) invisible boxes. The latter span any time period not covered by the user defined boxes. The observed states have a normal density, which is parametrized by the mean and variance of the expression values at the time interval defined by the time box (see Figure 3a and 3b). The hidden states have a uniform density. The length of the time boxes are equivalent to the expected duration per state (cf. eq 2). We restrict the topology to contain only successor- and self-transitions (see Figure 3B). We require the model to have unique start and end states and normalize the expected durations such, that their sum matches the length of the longest time course in the data set. The HMM has the following free parameters per state: The expected duration and the emission density parameters (see [2] for a detailed description of the model). In the following we detail how we derive these parameters from timeboxes and how queries can be defined using this model.

As depicted in Figure 2b, we can allow values out-side of , as long as the overall values of a time course fit the timebox. This can be obtained by modeling the value range covered by a timebox with one uni-variate Normal, where the mean defines the mid point of the value range of the timebox and the standard deviation is proportional to a half of the height of the box.

Additionally, we also want to account for variation along the time axis, in particular time courses with phase-shifts (cf. Figure 1b) and difference in speed (cf. Figure 1d). This can be achieved by modeling the time frame as an expected duration of a timebox. Such probabilistic timeboxes define a state with an uni-variate normal density emission in a lHMM [2]. In this model, we also need to assign states for time frames where no timebox is defined, such as the regions in the left of Figure 2b. Specifying states in such frames substantiates the assumption about *when *we expect the user defined normal models to occur. These hidden boxes can be interpreted as waiting timeboxes, where any observation is equally likely to occur.

More formally, a probabilistic timebox PTB is a state in a lHMM *λ *with an uni-variate normal density emission (see Figure 3B). This (*k*th) state is parametrized by the triplet (*p_k_*, *μ_k_*, *σ_k_*), where *p_k _*is the self transition probability or expected duration, and *N *(*μ_k_*, *σ_k_*) is the state emission density. Given the (graphical) parameters of a timebox (c.f. Figure 3A), the state parameters can be defined as,(2)(3)(4)

Note that the current choice of *σ_k _*places approximately 95.5% of the density mass in the interval , and that  is the expected duration of the timebox. The latter defines *p_k_*, which obeys a geometrical distribution [10]. The relation between transition probability and duration is detailed in [7]. The idea is to consider the probability of staying *n *times in state *s_i_*, before you make a transition to *s*_*i*+1_.

Hidden states, that have no graphical representation, model time frames between timeboxes. They are defined by a single parameter, their expected length, *p_l_*, and a uniform distribution U(*x_min_*, *x_max_*), where *x_min _*(and *x_max_*) are the minimum (and maximum) value in *O*. For two consecutive timeboxes, *j *and *j *+ 1, where , we have(5)

Time ranges before (and after) the first and last timeboxes *b*_1 _and *b_q _*represent special cases. We also include a state emitting a uniform distribution in these ranges when  and .

One additional requirement for querying with PTBs is a parameter *m*, which defines how many time courses should be returned by the query. Intuitively, the query works by estimating the time course likelihoods (**P**[*o_i_*|*λ*]) using the Forward-algorithm [7], followed by the selection of the *m *time courses with highest likelihoods. In particular setting *m *to the number of time courses will return all time courses as query result and setting *m *to 1 will return the time course with highest likelihood. More formally, given a full parametrized lHMM *λ *defined by the timeboxes (*b*_1_, ..., *b_q_*) and *m*, the probabilistic query is defined as,(6)

The main computational task behind the query is computing the log-likelihood of the time-courses for a given HMM. This has the computational complexity of *O*(*N · K · L*), where *N *is the number of time-courses, *K *the number of states (or time-boxes) and *L *the number of time points. Note that *K *and *L *are usually small (< 10) in this application, there-fore the running time only depends linearly on the number of samples *N*.

### Testing

#### Case Study - Yeast Sporulation

To show the usefulness of the probabilistic timeboxes in the analysis of gene expression, we perform a case study with the time courses of Yeast during sporulation [11] There, the expression values of approximately 6,200 genes have been measured through the course of seven time points (0 h, 0.5 h, 2 h, 5 h, 7 h, 9 h and 11 h). The biological process is formed by a cascade of transcriptional events, which can be subdivided in three main consecutive events: DNA replication, meiosis and spore maturation. We pre-processed the data, as described in [2], to discard time courses with a large number of missing values or with small temporal expression change, which resulted in a data set with 1,171 genes. In [11], authors hand selected seven groups of genes as prototypes of interesting expression patterns. For example, genes with an increase in expression in early, middle or late time points. The mean expression value of each group was taken, and a nearest neighbor approach based on the correlation coefficient was used to assign other genes to each of the expression patterns.

**Figure 4:**
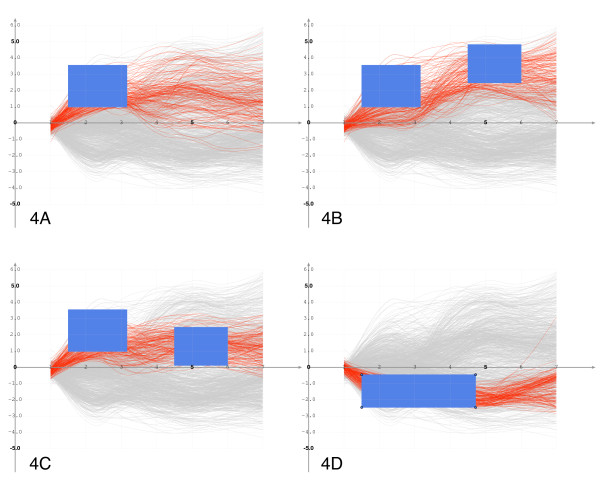
**Yeast sporulation case study**. We depict query results from a Yeast sporulation data set. In the first query (a), we have 150 genes with early over expression pattern. This query is made more specific by creating a new timebox (b). Now, it returns genes that have a sustained over-expression pattern. By applying a move operation on the second timebox, we query for genes with a over-expression followed by expression decrease (c). We can look for further patterns by deleting the second timebox and move the first timebox down to obtain genes with under-expression patterns(d).

We show here, how such exploratory analysis of temporal expression patterns, can be interactively performed with the PTBs. In the following we refer to a pattern of over-expression, which is visible in data and can be scored by our model, simply as over-expression. For example, to find all genes with early over-expression patterns, we simply need to draw a timebox in the upper left side of the canvas and set the query stringency to 150 genes (Figure 4a). Note that all time courses display high expression at early time points but distinct expression patterns at later time points. A functional analysis of the genes indicates that the query contains genes mostly related to metabolism and meiosis. To refine the query, we can create an additional PTB, now at the upper-right part of the canvas (Figure 4b). This query will return all genes with an over-expression pattern, which is sustained over time. Functional analysis of these genes indicates a relation to meiosis, which is in accordance with the functional analysis performed in [11]. We can then modify the query, by moving the second timebox toward zero expression values, therefore querying genes with a over-expression followed by an expression decrease (Figure 4c). These genes, which are involved in metabolism, are activated as a reflex of the nitrogen starvation caused by the sporulation medium [11]. The user can perform further operations on the timeboxes to find other expression patterns of interest. Deleting the second PTB and moving the first one down returns a query with genes displaying under-expression pattern (Figure 4d). These genes are mostly related to the ribosome, which has been interpreted as effect of the cessation of growth under the nitrogen starvation [11]. This example displays how a user can easily perform an exploratory analysis of temporal expression patterns with PTBs and all main features provided by pGQL.

### Implementation

The software is implemented in Python and Tkinter using the GHMM library [5] and was tested on Linux and Mac OS X, but works on other modern operating systems including Windows as well. It is licensed under the GPL. The software is available from our web site http://algorithmics.molgen.mpg.de/Software/pGQL/.

The tool automatically inserts (and removes) hidden states into (from) regions, where the user has not (has) defined a PTB for a state corresponding to a normal emission density. These hidden states correspond to uniform emission densities. While drawing and editing PTBs consistency constraints are automatically checked and create a frame for specifying useful models. In particular pGQL checks, that users draw boxes with a minimum of 1.0 expected duration, since we do not assume any interpolation model on the discrete data. Users are also automatically guided to never produce overlapping states or states extending beyond the graph display. When-ever a query is graphically modified pGQL automatically recomputes the query and updates the result. It is possible to display PTBs on top of the time course data for query redefinition or below to inspect query results. pGQL also implements deterministic timebox queries to enable direct comparisons of the methods by switching between query types on the fly.

#### Functional Analysis

An important method for the biological analysis of a group of genes, as the one returned by a query, is the so called enrichment functional analysis [12]. This method uses databases with functional annotation of genes, such as Gene Ontology [13] to search for functional groups, whose genes are also within the query result. pGQL provides an out-link to the G: Profiler tool [12] to perform an enrichment functional analysis based on the current working query.

## Discussion

The PTB based graphical query approach enables users to phrase their hypotheses in a fashion many are already familiar with, i.e. defining normal distributions in the value domain and placing them as expected durations. Uncertainty is naturally incorporated in such models, while the user is not required to bother with the formalisms, but can simply use an interactive tool, that alleviates the usage of these models. The specificity of queries can easily be in-creased by adding more PTBs to a query. It would be interesting to combine pGQL with formal queries known from other field s to benefit from many deterministic queries as well. It is a future research problem how to translate such queries into one common graphical query interface. The only deterministic query pGQL currently provides is of the basic time box query type [4]. Although beyond the scope of the present article, it would be interesting to perform a full usability study of our tool in the future to conduct a user related performance evaluation as well.

The interpretation of query results from pGQL is straightforward: The result set will contain the *m *top ranking time courses, where obviously the highest scores will be assigned to time courses that lie centrally to the query. In contrast deterministic queries do not have a notion of rank as their scoring is binary.

We could in principle approximate the behavior of deterministic boxes by imposing peak like boxes on time periods. From a statistical point of view this is equivalent to overfitting the query model on the time dimension. Since the measurements will be taken in units of time, it generally doesn't make much sense from a modeling perspective to expect any measurement to last shorter than a a full time unit. Thus pGQL requires at least a unit of time for each timebox. This choice leads to query models, that are robust to time delays, since the self-transition probability in the HMM will not be forced to be infinitesimally small. If a user has a strong belief in values taking place in an exact, predefined time period he might get additional time courses returned from the query model, that exhibit similar behavior delayed in time. The ranking of time courses is one way to re ne the selection of such a result set: Time courses that adhere to the query during the exact time periods of the probabilistic boxes, should always be contained in the results, as they will be ranked highly by the query model.

Deterministic timeboxes could in principle be made approximate by requiring only a certain fraction of measurements to lie within a box. This shifts the problem to determining what this fraction really is. It is unclear how well this concept could capture common time course characteristics we mentioned in the Background section.

An example (again on the yeast sporulation data) demonstrating the robustness of the probabilistic queries is shown in Figure 2b: Despite of outliers on time points four and five the query result shows time courses displaying strongly similar behavior. The ex-act same graphical query in the deterministic setting shown in Figure 2a returns only a subset of the probabilistic query result, which is owed to the sensitivity to outliers. Moreover the result contains a few time courses, that do not seem to fit the overall trend specified by the query well. The latter is an artifact owed to the over fitting in time.

## Conclusions

Probabilistic timeboxes introduced new semantics to timeboxes proposed by [4]. Graphical queries are interactively created and manipulated by enabling users to draw, delete, move and resize any number of PTB's with respect to the incorporated constraints of this approach. This allows for easy and comfortable definition of meaningful, flexible queries even in complex data analysis scenarios. Probabilistic models, in particular in use for gene expression data, are known to be much more appropriate and flexible than their deterministic counter parts [2,14-16]. The ease of the graphical definition encapsulates a sound method for query definition, requiring no in-depth knowledge of the underlying probabilistic methods to use this tool. We have demonstrated the method on a real world data set of yeast sporulation. This case study also revealed typical advantages our method has over deterministic methods. We contrasted the PTBs with the original timeboxes and shown how they cope better with many properties of gene expression time courses. pGQL can be of great help in finding interesting clusters in time course data.

A straight-forward extension to our method would be modeling cyclic data. All that is needed for this is to insert a transition from the end state to the start state and specify the transition probability favoring more or fewer cycles. Then we expect cyclic or periodic data to benefit from queries with probabilistic timeboxes just as well. GHMM supports cyclic queries already.

## Competing interests

The authors declare that they have no competing interests.

## Authors' contributions

RS implemented the graphical tool and drafted the manuscript. IGC and AS implemented the probabilistic models and contributed to the manuscript. AS conceived the study initially. All authors read and approved the final manuscript.
